# A novel non-invasive approach monitoring skeletal stem cell function through ^18^F-Pentixafor PET-CT

**DOI:** 10.1016/j.jot.2026.101129

**Published:** 2026-05-20

**Authors:** Zan Li, Dongsheng Zhang, Dilibire Adili, Jie Han, Zhenwen Xie, Qianhe Xu, Lixin Tian, Guangfa Wang, Guolin Wang, Peipei Wang, Matthew B. Greenblatt, Xinhui Su

**Affiliations:** a Department of Nuclear Medicine & PET Center, The First Affiliated Hospital of Zhejiang University School of Medicine, Hangzhou, 311113, China; b Cancer Research Center, School of Medicine, Faculty of Medicine and Life Sciences, Xiamen University, Xiamen, Fujian, China; c The State Key Laboratory of Fluid Power and Mechatronic Systems, College of Mechanical Engineering, Zhejiang University, Hangzhou, China; d Department of Pathology and Laboratory Medicine, Weill Cornell Medicine, New York, NY, 10065, USA; e Research Division, Hospital for Special Surgery, New York, NY, 10065, USA

**Keywords:** ^8^F-pentixafor, Bone regeneration, CXCR4, Fracture healing, PET imaging, Skeletal stem cells

## Abstract

**Background:**

Fracture nonunion remains a major clinical challenge and is closely associated with dysfunction of osteogenic-lineage cells and their progenitors, skeletal stem cells (SSCs). However, conventional clinical approaches lack the specificity required to assess SSC functional status *in vivo*. Radiotracer-based positron emission tomography-computed tomography (PET-CT) provides an opportunity for noninvasive functional imaging of SSCs.

**Methods:**

We first integrated RNA sequencing with FDA-approved radiotracers, to demonstrate *Cxcr4* expression in murine SSCs, sorted by fluorescence-activated cell sorting (FACS), compared to non-stem skeletal cells (NSCs). Furthermore, we isolated SSCs and cultured them *in vitro* with CXCR4-agonist or antagonist to evaluate the role of CXCR4 in SSC differentiation and mineralization. We utilized ^18^F-Pentixafor, a clinically available CXCR4-targeting radiotracer, was used for *in vivo* PET-CT imaging to monitor SSCs in both murine models and human patients. We then generate bi-cortical femoral fracture models, take advantage of three-point bending tests and combined ^18^F-Pentixafor-based PET-CT to assess the correlation of ^18^F-Pentixafor uptake and biomechanical tests in bone regeneration. Bone phenotype of murine models was analyzed using micro-CT and histological studies. And we finally involved a case-report of a young patient suffering hamulus fracture to evaluate the role of ^18^F-Pentixafor-based PET imaging in assessing bone regeneration and assist in decision on clinical administration to approve the translational potency of ^18^F-Pentixafor-based PET imaging.

**Results:**

By integrating SSC RNA sequencing with FDA-approved or radiotracers in III/IV phase clinical trials, we identified CXCR4 as a promising diagnostic target and evaluated SSC activity using ^18^F-Pentixafor, a clinically available CXCR4-targeting radiotracer. Bulk RNA sequencing demonstrated markedly enriched *Cxcr4* expression in SSCs relative to non-stem skeletal cells (NSCs), a finding supported by CXCR4 co-localization with SSC markers in fracture callus and by ^18^F-Pentixafor accumulation in the human growth plate. CXCR4 signaling also reflected SSC function, as CXCR4 agonist stimulation enhanced SSC differentiation and mineralization *in vitro*, whereas antagonists impaired these processes and delayed fracture healing *in vivo*. In mice, ^18^F-Pentixafor uptake was higher in young than aged femoral metaphyses, consistent with reduced tracer uptake in the femoral heads of aged human subjects. Compared with ^18^F-FDG and ^18^F-NaF, ^18^F-Pentixafor more accurately predicted biomechanical properties of fracture repair. A prospective case further demonstrated clinical feasibility in monitoring hamulus healing.

**Conclusion:**

Together, these findings establish CXCR4-targeted ^18^F-Pentixafor PET imaging as a sensitive, non-invasive tool for visualizing SSC enrichment and function to aid in evaluating bone regeneration potential.

**Translational potential of this article:**

This study facilitates the translation of fundamental skeletal stem cell (SSC) biology into clinical decision-making by repurposing the CXCR4-targeting radiotracer ^18^F-Pentixafor for functional imaging of bone regeneration. We not only elucidate the role of CXCR4 in SSC function but also establish that CXCR4 as a novel target for *in vivo* SSC monitoring. These findings suggest that ^18^F-Pentixafor-based PET imaging targeting CXCR4-expressing SSCs may enable early risk stratification for fracture nonunion, improve the assessment of age-related or disease-associated impairments in bone regeneration, and support therapeutic decision-making in clinical practice.

## Graphical abstract

**Figure:**
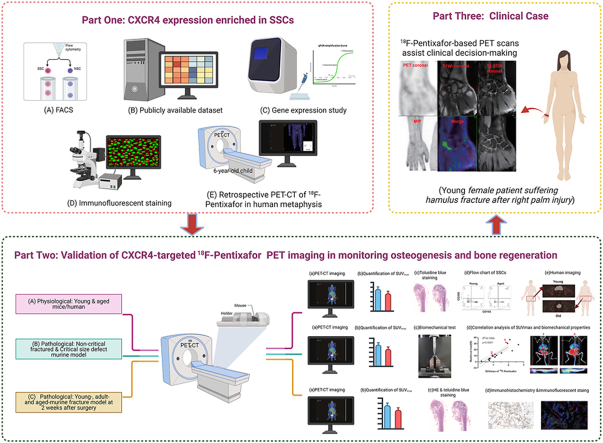

## 1 Introduction

Fracture nonunion is an uncommon but devastating complication, as failure of the bone to heal disrupts mobility, impairs function, and often necessitates complex reconstructive procedures [[1], [2], [3]]. These consequences significantly compromise quality of life and escalate healthcare costs [4,5]. Demographic information and patient history have only a limited ability to predict which patients will ultimately display nonunion when presenting with a fracture. Conventional imaging modalities (e.g., CT, MRI, DEXA) quantify structural bone properties but fail to capture the dynamic cellular processes underlying bone formation and repair [6]. Bone turnover markers, such as procollagen type 1 N-terminal propeptide (P1NP) and bone-specific alkaline phosphatase (b-ALP), partially reflect systemic bone formation and resorption but do not accurately represent localized osteogenesis at the fracture site [7].

Molecular imaging techniques, especially positron emission tomography-computer tomography (PET-CT), offers a promising solution by the ability to non-invasively assess osteoblastic-lineage cell abundance or function [8,9]. To date, applications of PET-CT in bone-regeneration monitoring have relied mostly on ^18^F-FDG and ^18^F-NaF probes. However, ^18^F-FDG uptake reflects generalized glucose metabolism, and in the context of fracture healing this can include contributions from infiltrating non-skeletal cell types, while the ^18^F-NaF uptake indicates osteoid deposition rather than the abundance or function of osteoblasts. Thus, neither ^18^F-FDG nor ^18^F-NaF provides sufficient specificity for evaluating SSC amount or activity, which ultimately impedes the ability to predict fracture repair outcomes to guide clinical management of at risk patients.

SSCs, defined in mice by CD51 and CD200 expression [10,11], are the major progenitor source for osteoblasts during bone development, homeostasis, and regeneration [10,12,13]. Intrinsic aging of SSCs can lead to reduced osteogenic capacity contributing to aging associated reductions in fracture healing [14,15]. Decline in SSC function is a major driver of impaired fracture healing and nonunion [[16], [17], [18]]. Despite progress are made in characterizing SSCs and their lineage diversity [13,19,20], there are currently no tools capable of noninvasively monitoring SSC activity or abundance *in vivo* [11]. This gap impedes early diagnosis of SSC dysfunction, assessment of regenerative therapies, and personalized management of bone disorders.

To address this gap, we screened published transcriptomic datasets and cross-referenced candidate surface markers with FDA-approved or PET tracers in III/IV phase clinical trials [19]. CXCR4 emerged as a promising target based on its high expression in long-bone SSCs and its established roles in stem cell homing, osteogenic differentiation, and bone remodeling [21,22]. The clinical radiotracer ^18^F-Pentixafor, a high-affinity CXCR4 ligand, is a radiotracer for oncologic imaging, however it has yet to be considered for bone biology applications [23,24].

Here, we integrate transcriptomics, functional assays, preclinical imaging, and human PET-CT data to evaluate CXCR4-targeted ^18^F-Pentixafor PET-CT as a tool for monitoring SSC activity *in vivo*. We demonstrate that ^18^F-Pentixafor uptake reflects SSC abundance and osteogenic potential during bone development, aging, and fracture repair, and that it outperforms ^18^F-FDG and ^18^F-NaF in predicting fracture-healing outcomes. These findings reveal a new application for CXCR4-directed PET imaging in skeletal regeneration.

## 2 Materials and methods

### 2.1 Animals and the bi-cortical femoral fracture model and critical-sized defect model

Mice were housed and bred in a barrier facility and were fed ad libitum chow. Housing followed a standard day-night lighting cycle. Femoral fracture was performed as previously described [25,26]. In brief, 3-month-old male mice were anesthetized with isoflurane, shaved and maintained a left-lateral position. A 3-mm incision was made on the anterolateral side above the longitudinal axis of femur. The patella was exposed and dislocated to prevent injury to the patellar tendon. A 27-gauge syringe needle was inserted from intercondylar fossa along the longitudinal axis of the femur into the marrow cavity. The needle was then removed and the spatium intermusculare was blunt dissected to expose the midshaft of femur. The sciatic nerve was dissected and protected during surgery. The femur was then bisected at the midshaft with a dental saw with a thin-diamond cutting wheel. A 25-gauge needle was then relocated into the marrow cavity to stabilize the fracture sites and needle was trimmed to avoid secondary injuries due to the needle projecting into the stifle joint. For CXCR4-antagonist local treatment, 50ug Plerixafor (half-time 4.6 h *in vivo*) were dissolved in Matrigel (Corning) and administered directly in the fracture line and suture is made after solidification of Matrigel. For critical-sized defect, a cylindrical defect in femoral diaphysis with >2 mm in height was selected as critical-sized defect. Intramedullary fixation was performed before the closure of the wound [27]. The patellar tendon was relocated, and muscles near the surgical site were reduced. The skin incision was closed with absorbable sutures. Analgesic administration, meloxicam 4 mg/kg/d was performed subcutaneously as described above.

### 2.2 Key reagents and resources

Key reagents and resources were listed in Table 1 as below.

**Table 1:** Key reagent or resources.

REAGENT or RESOURCE	SOURCE	IDENTIFIER
**Rabbit Anti-Cxcr4**	**abcam**	**Cat#: ab124824**
**Rat Anti-CD51**	**Invitrogen**	**Cat#: 14**–**0512**–**82**
**Goat anti-Rabbit IgG (H + L) Cross-Absorbed Secondary Antibody, Alexa Fluor^TM^ 488**	**Invitrogen**	**Cat#:A11008**
**Donkey anti-Rat IgG (H + L) Highly Cross-Absorbed Secondary Antibody, Alexa Fluor^TM^ 594**	**Invitrogen**	**Cat#: A21209**
**Donkey Serum**	**abcam**	**Cat#: ab7475**
**Alizarin red S**	**Sigma**	**Cat#: A5533**
**Toluidine blue**	**Sigma**	**Cat#: T3260**
**Alkaline Phosphatase Assay Kit**	**Beyotime**	**Cat#: P0321S**
**Collagenase P**	**Roche**	**Cat#: 11213857001**
**Dispase II**	**Roche**	**Cat#: 04942078001**

### 2.3 ^18^F-pentixafor, ^18^F-NaF and ^18^F-FDG PET/CT acquisition and imaging

^18^F-NaF and ^18^F-FDG were prepared and related PET-CT were performed as previously described [[28], [29], [30], [31]]. ^18^F-Pentixafor was prepared in our center with IV certificates for the use of radioactive drugs. Briefly, the pentixafor reagent kit was purchased from Beijing PET Technology Co. Ltd. (Beijing, China). Fluorine-18 was produced on site using a Siemens Eclipse cyclotron (Siemens Medical Solutions, Knoxville, TN 37,932, USA). The radiochemical synthesis of 18F-Pentixafor was performed in an AllInOne® synthesis module (Trasis, Ans, Belgium). Subsequent quality control was performed using validated analytical procedures. The radiochemical purity of ^18^F-Pentixafor was over 95%. For patient scanning, intravenous injection of ^18^F-Pentixafor at doses of 3.7 to 4.44 MBq/kg was administered. 60 min after ^18^F-Pentixafor injection, patients underwent a PET/CT scan (Biograph version; Siemens, Germany) from top skull to mid-thigh. All data were transferred to a Siemens workstation (syngo.via Client 4.1) and reconstructed using a MedExsystem nuclear medical information system (MedEx Technology Limited Corporation, Beijing, China).

For animal scanning, different ages of C57BL/6 J male mice without fracture were used for imaging experiments. Mice were anesthetized with 1.5–2% isoflurane inhalation. Mice then received an intravenous injection of 200 μL 3.7 ± 0.37 MBq/mouse ^18^F-Pentixafor. Micro PET/CT (Sirius, Pingseng, China) and scanning was performed approximately 60 min post-injection of ^18^F-Pentixafor. Standard CT scanning protocols were used for image acquisition, and a 3D Ordered Subset Expected Maximum (OSEM-3D) algorithm was used to reconstruct the image. The regions of interest (ROIs) corresponding to areas of obvious radioactivity accumulation in the metaphysis or fracture callus were manually drawn. Background ROIs with a similar diameter were drawn in the contralateral thigh muscle. The maximum standard uptake value (SUV_max_) and percentage injected dose per gram of tissue (ID%/g) for each ROI were measured before statistical analysis.

### 2.4 Image interpretation

Analysis of PET-CT images were independently performed by two experienced nuclear medicine physicians. They were blinded to the clinical/preclinical data and pathological results and independently analyzed all images. Any discordant results were resolved by consensus. Focal ^18^F-Pentixafor, ^18^F-NaF and ^18^F-FDG accumulation showing activity higher than background after accounting for physiological uptake of that tissue, were considered positive. ^18^F-Pentixafor, ^18^F-NaF and ^18^F-FDG uptake of positive area was semi-quantified by the maximal standardized uptake value (SUV_max_). The region of interest (ROI) was drawn to determine the SUV_max_.

### 2.5 Cell isolation and fluorescence-activated cell sorting (FACS)

Cell isolation and FACS were performed as previously described [13,19,20]. In brief, the fracture callus was isolated 15-day post-surgery as a site enriched for SSCs mediating the fracture response [13], subjected to physical disruption before digestion with Collagenase P (0.1% w/v) and Dispase II (0.2% w/v) to generate a single cell suspension as previously described [13,32,33]. After digestion, cells were filtered through a 70-μm filter into a conical tube before centrifugation, and the prepared single-cell suspensions were washed twice with ice-cold FACS buffer (2% sera + 1 mM EDTA in PBS) followed by Fc blocking buffer (1:100 dilution; Cat No. 553142, BD bioscience) incubation. Primary antibodies were prepared in Brilliant Stain Buffer (1:100 dilution; Cat No. 563794, BD bioscience). Cells were incubated in the dark for at least 0.5h on ice with primary antibody solution and washed 2 times with ice-cold FACS buffer. FACS was performed using a Becton Dickinson Aria Fusion (BD Bioscience). Beads (01-3333-42, Invitrogen) were used to set initial compensation. Fluorescence minus-one (FMO) controls were used for additional compensation to assess background levels of each stain. Gates were drawn as determined by internal FMO controls to separate positive and negative populations. Cells were collected in α-MEM supplied with 20% fetal bovine serum before cultured on a 12-well plate. Data were analyzed with FlowJo (v10.8.1).

### 2.6 FACS antibodies

Antibodies for FACS of murine samples included CD31 (MEC13.3, BD Bioscience), CD45 (clone 30-F11, BD Bioscience), Ter119 (clone TER-119, BD Bioscience), Ly-51 (clone 6C3, BD Bioscience), THY1-1.2 (clone 53-2.1, BD Bioscience), CD51 (551187, RMV-7, BD Bioscience), CD200 (clone OX-90, BD Bioscience) and CD105 (clone MJ7/18, BioLegend).

### 2.7 Bulk RNA-seq data analysis

Bulk RNA-Seq analysis on FACS-isolated SSCs were performed as previously described [13,19]. Briefly, total RNA was freshly extracted from 2 × 10^4^ FACS-isolated cell populations of wild type mice using the RNeasy Plus Micro Kit (Qiagen, Cat# 74034). RNA quality and quantity were verified using an Agilent 2100 Bioanalyzer system. cDNA libraries were generated with the Illumina SMART-Seq v4 Ultra Low Input RNA Kit and the Nextera XT DNA Library Preparation Kit, followed by sequencing on a NovaSeq 6000 sequencer (Illumina) to generate paired-end 150-bp reads. Raw sequencing reads in BCL format were converted to FASTQ files and demultiplexed using bcl2fastq v2.20 (Illumina). Adapters were trimmed using Cutadapt (v1.18). RNA reads were aligned and mapped to the GRCm38 mouse reference genome using HISAT2 (v2.2.1). Transcriptome reconstruction was featureCounts (v2.0.3) from the Subread package. Transcript abundance was quantified by edgeR (v4.7.3) in fragments per kilobase of exon per million mapped reads (FPKM). Principal component analysis (PCA) was performed using the ggbiplot (v0.6.2) package to visualize global gene expression patterns across samples. Hierarchical clustering was conducted on standardized expression data to assess sample similarity. Differential expression analysis was carried out using DESeq2 (v1.49.2), with normalization of raw counts and dispersion estimation based on a negative binomial model. Genes with P < 0.05 and |log_2_FoldChange| > 1 were considered significantly differentially expressed. Normalized read counts were used to generate heatmap plots. Data were visualized as a heatmap with hierarchical clustering in R (v4.2.0) using the pheatmap (v1.0.13) package.

### 2.8 Three-point-bending assay of fracture callus

A three-point bending test was conducted to evaluate the bending strength and stiffness of the mouse fracture callus [34,35]. In brief, murine femur were collected at 4 weeks after fracture surgery as the callus is essentially fully mineralized at that time [36]. Mechanical testing was performed using a universal testing machine (UTM-2203; Shenzhen Suns Technology Stock Co., Ltd., Shenzhen, China). The support span was set to 10 mm. After preprocessing, each bone specimen was positioned on the two supports with its longitudinal axis aligned parallel to the span, ensuring that the flatter cortical surface faced upward to provide a stable and reproducible loading condition. A loading indenter with a rounded tip (R5) was placed at the midpoint of the span, maintaining line contact with the bone surface.

The test was performed in displacement-control mode at a loading rate of 0.5 mm/min to ensure a quasi-static loading condition. Throughout the experiment, the load and displacement were recorded synchronously, and these data were subsequently used to calculate the maximum failure load.

### 2.9 Isolation of mouse SSCs, cell culture, differentiation and cell-binding assays

Mouse SSCs isolation and *in vitro* differentiation assays were performed as previously described [13]. After 10-day differentiation induction, SSCs were subjected to Alizarin Red Staining (ARS) and an Alizarin Red Assay (ARA) as previously described [25,37]. Cell binding assay was performed based on previous report [38]. In brief, FACS-isolated SSCs and NSCs were washed with ice-cold PBS twice before incubation with ^18^F-Pentixafor for 60 min. After incubation, serial-diluted amount of cells (indicated in Supplementary Fig. 1B) were washed twice with ice-cold PBS in 200uL EP tube and concentrated to the cell pellet before PET scan.

### 2.10 Quantitative PCR

Quantitative polymerase chain reaction (qPCR) studies were performed as previously described [19,20,26]. mRNA expression levels were normalized with murine *Gapdh*. Samples were evaluated for differences in the transcription levels of osteogenic markers. The primers used for qPCR are listed below.

- *Gapdh* Forward: CATCACTGCCACCCAGAAGACTG
- *Gapdh* Reverse: ATGCCAGTGAGCTTCCCGTTCAG
- *Cxcr4* Forward: GACTGGCATAGTCGGCAATGGA
- *Cxcr4* Reverse: CAAAGAGGAGGTCAGCCACTGA

### 2.11 Histological assessment

Histological examination of bone phenotypes were performed as previously described [39,40]. For histological evaluation of fracture callus, femur callus samples were decalcified for 3 weeks before processing paraffin embedding. Blocks were sectioned at 5 μm thickness followed by H&E staining and toluidine blue staining as previously described [33].

### 2.12 Immunohistochemistry

In brief, frozen samples were thawed and rehydrated at room temperature with PBS. Followed by permeabilization with 0.3% Triton X-100 (Sigma) and blocked for 0.5h with 5% donkey serum. Primary antibodies (1:200-1:500) were freshly prepared in 0.3% Triton X-100 and samples were incubated with primary antibodies overnight at 4 °C. A 3-time wash with PBS was performed to remove excess antibodies. Secondary antibodies (1:1000 dilution) were added to samples and incubated for 1 h. Samples were then washed three times with PBS. Slides were mounted with ProLong Gold antifade reagent with DAPI (Invitrogen, cat. P36931). Imaging was performed with a Leica TCS SP8 DLS laser scanning confocal microscope. All data were processed using Leica Application Suite Advanced Fluorescence 3.0.0 build 8134 software.

### 2.13 Micro-computed tomography (μCT) imaging and data analysis

Micro-computed tomography (μCT) was conducted using SkyScan 1272 (Bruker, Germany) as previously described [41]. Femurs were harvested and scanned at 10 μm resolution. For analysis of femoral trabecular bone mass, NR econ program was utilized to define 1 mm wide contoured regions of trabecular bone starting 500 microns above the proximal end of femoral growth plate. Cortical bone regions of interest (ROIs) were defined as 1 mm in height and centered on the midshaft. Binarization was performed using a constant threshold (80-255). Cortical and trabecular thickness (Cs. Th, Tb. Th), trabecular number (Tb. N), trabecular separation (Tb. Sp) and bone volume/tissue volume (BV/TV) were calculated by contouring the corresponding regions of the scan slices through cTAn software. 3D reconstruction images were generated by stacking the contours of 2D images. μCT analysis was conducted by an individual researcher blinded to the genotype of each mouse.

### 2.14 Docking analysis

The X-ray crystal structures of CXCR4 (PDB: 9MEU) were retrieved from the Protein Data Bank. The protonation state of all the compounds was set at pH = 7.4, and the compounds were expanded to 3D structures using Open Babel [42]. AutoDock Tools (ADT3) were applied to prepare and parametrize the receptor protein and ligands. The docking grid documents were generated by AutoGrid of sitemap, and AutoDock Vina (1.2.0) was used for docking simulation [43,44]. The optimal pose was selected to analysis interaction. Finally, the protein-ligand interaction figure was generated by PyMOL. The CXCR4 protein is represented as a slate cartoon model, ligand is shown as a cyan stick, and their binding sites are shown as magentas stick structures. Nonpolar hydrogen atoms are omitted. The hydrogen bond, ionic interactions, and hydrophobic interactions are depicted as yellow, magentas and green dashed lines, respectively.

### 2.15 Patient selection criteria, health condition and informed consent

The retrospective cohort enrolled 37 patients. Inclusion criteria: i. Age 18 to 80 years diagnosed with; ii. Absence of congenital bone disorders; iii. Absence of other conditions causing femoral bone lesions; iv. No surgical history of femoral head; v. Absence of concomitant malignancy or other comorbidities affecting bone quality. Exclusion criteria: Age <18 or >80 years.

For prognostic evaluation, only one patient is included in this study and demonstrated as a case-report. Patient diagnosed with limb fracture, including wrist bone fracture was included for the evaluation of the diagnostic value of ^18^F-Pentixafor on monitoring SSC. The enrolled patient were diagnosed with hamate fracture with normal blood routine, liver and kidney function without history of congenital skeletal disorders. The enrolled patient provided written informed consent.

### 2.16 Statistical analysis

**Fig. 1:**
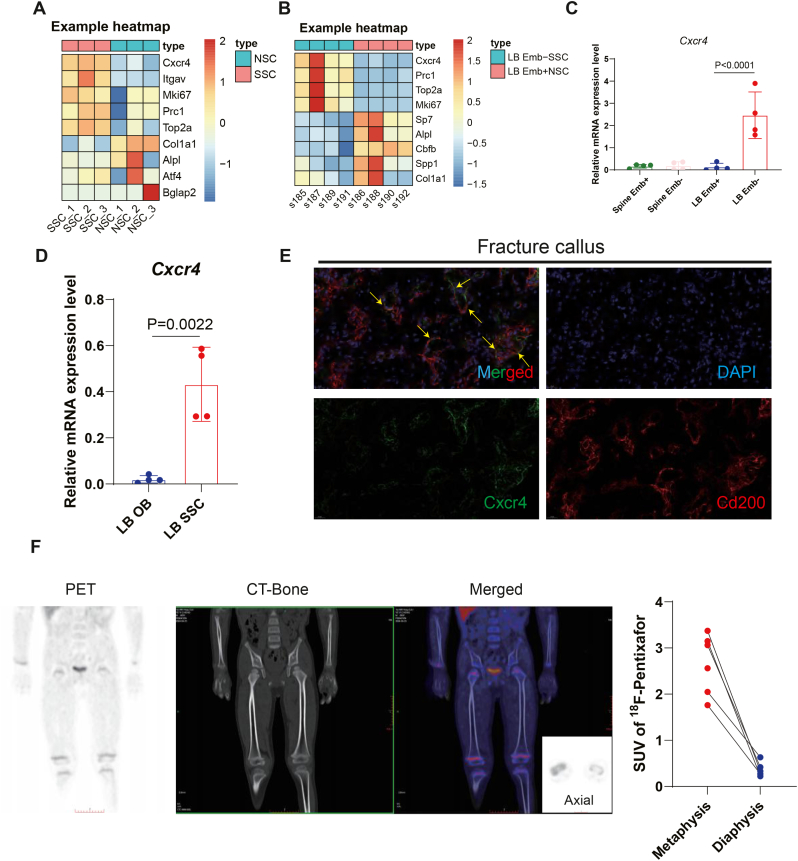
Identification of CXCR4 as a marker of SSCs enabling CXCR4-targeted PET-CT monitoring (A) Heatmap showing representative differential genes between murine SSCs and NSCs (n = 3 per group). (B) Heatmap showing differential genes between murine Emb- SSCs and Emb + subsets by reanalyzing publicly available dataset (GSE230852 and GSE230724, n = 4 per group). (C) Relative expression level of *Cxcr4* in spinal or long bone (LB) Emb- SSCs and Emb + subsets (GSE230852 and GSE230724, n = 4 per group. Results are presented as mean ± SD. Statistical analysis: One-way ANOVA with Tukey's multiple comparisons test). (D) qPCR results showing expression level of *Cxcr4* in long bone SSCs and BMSC-differentiated osteoblasts (n = 4 for each group). (E) Representative immunofluorescent staining in murine fracture callus showing co-localization of Cxcr4 and Cd51 in murine fracture callus. (F) Representative human (6-year-old child) PET-CT scan showing ^18^F-Pentixafor uptake in growth plate area and quantification of SUV_max_ of ^18^F-Pentixafor uptake in metaphysis area versus diaphysis area. Scale bar shown as labeled.

**Fig. 2:**
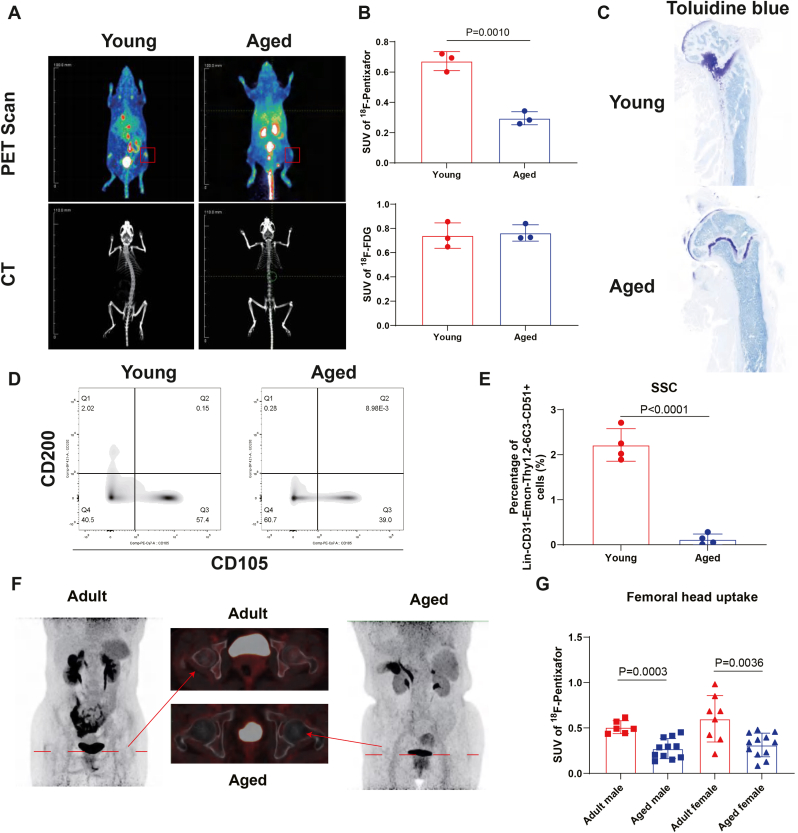
Reduced ^18^F-Pentixafor uptake reflects age-related loss of SSCs in long bone metaphyses (A) Representative PET-CT results showing ^18^F-Pentixafor uptake in metaphysis of young and aged mice. Scale bar shown as labeled. (B) Quantification of SUV_max_ in metaphysis area of ^18^F-Pentixafor (upper), ^18^F-FDG (lower) and (C) representative Toluidine blue (right) staining showing microstructure of metaphysis area. Results are presented as mean ± SD. Statistical analysis: unpaired *t*-test. *P* values were directly labeled above the dot plots for identification of significance. (n = 3 per group) (D) Representative flow chart showing SSC abundancy collected from lone bone of young and aged mice and (E) quantification of SSC abundancy (n = 4 per group, Results are presented as mean ± SD. Statistical analysis: 2-way ANOVA. *P* values were directly labeled above the dot plots for identification of significance). (F) Representative PET-CT results showing ^18^F-Pentixafor uptake in femoral metaphysis area of adult and aged female patients and (G) quantification of SUVmax of ^18^F-Pentixafor uptake in femoral metaphysis area of adult and aged male, female patients, respectively (adult group is defined as patients aging from 16 to 40 years old and aged group is defined as patients aging more than 60 years old. n = 6 for adult male patients, n = 11 for aged male patients, n = 8 for adult female patients, n = 12 for aged female patients. Results are presented as mean ± SD. Statistical analysis: One-way ANOVA with Tukey's multiple comparisons test. *P* values were directly labeled above the dot plots for identification of significance).

**Fig. 3:**
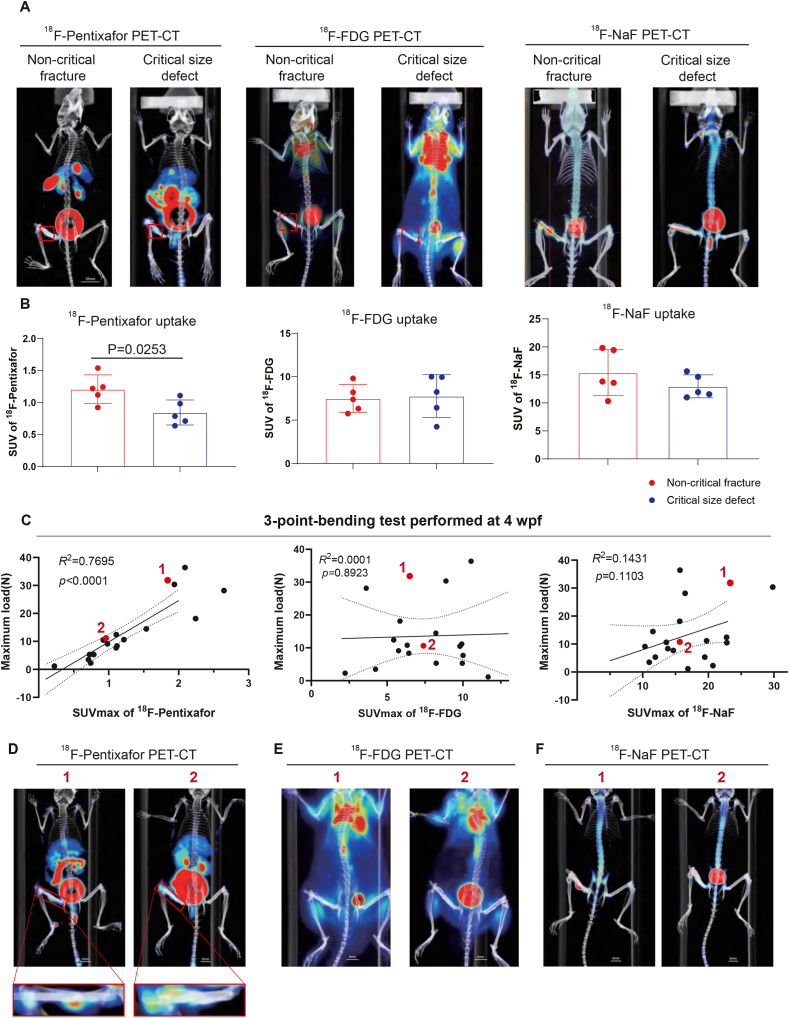
CXCR4 levels correlate with SSC function, and CXCR4-targeted radiotracer signal positively-correlates with biomechanical outcomes in murine fracture model (A) Representative PET-CT images showing ^18^F-Pentixafor, ^18^F-FDG and ^18^F-NaF uptake in fracture callus of non-critical fracture and critical size defect murine model (3-month-old, n = 5 per group) at 3 weeks after surgery. Scale bar = 10 mm. (B) Quantification of SUV_max_ in fracture callus of ^18^F-Pentixafor, ^18^F-FDG and ^18^F-NaF. Results are presented as mean ± SD. Statistical analysis: Student's t test. (C) Correlation analysis of maximum load from 3-point-bending test and SUV_max_ in fracture callus of ^18^F-Pentixafor (left), ^18^F-FDG (middle) and ^18^F-NaF (right), respectively (n = 10 per group). The red dot (1, 2) represents 2 mice that underwent non-critical sized femoral fractured model. ^18^F-Pentixafor, ^18^F-FDG and ^18^F-NaF PET-CT images are displayed in (D-F). WPF: Week Post-Fracture. *R*^*2*^ and *p* value directly labeled above the plots for identification of correlation and significance, respectively. (D-F) Representative PET-CT images showing ^18^F-Pentixafor, ^18^F-FDG and ^18^F-NaF uptake in the fracture callus of 2 mice that underwent a non-critical sized femoral fracture, 1 represents a murine model with higher ^18^F-Pentixafor uptake in callus area, bigger mineralized callus formation and stronger maximum load, while 2 represents a murine model with lower ^18^F-Pentixafor uptake in callus area, smaller mineralized callus formation and weaker maximum load. Scale bar = 5 mm.

**Fig. 4:**
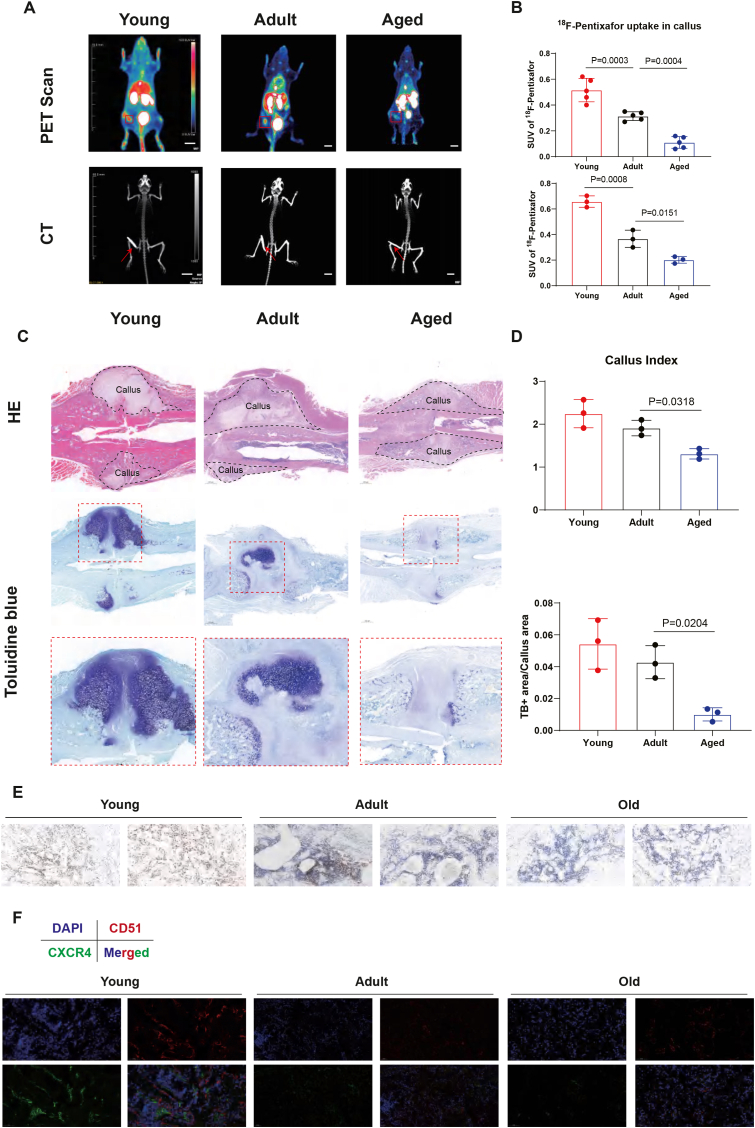
Decreased ^18^F-Pentixafor uptake in the aged fracture callus corresponds to reduced Cxcr4^+^ SSCs and impaired fracture healing (A) Representative PET-CT images showing ^18^F-Pentixafor uptake in fracture callus of young (1 month old), adult (3 month old) and aged (20 month old) mice at 3 weeks after surgery. Scale bar = 10 mm. (B) Quantification of SUV_max_ of ^18^F-Pentixafor in fracture callus of young, adult and aged mice at 3 (upper) and 4 weeks (lower) after surgery (n = 5 for 3 weeks, n = 3 for 4 weeks. Results are presented as mean ± SD. Statistical analysis: One-way ANOVA. *P* values were directly labeled above the dot plots for identification of significance.). (C) Representative HE staining (upper) and toluidine blue staining (middle and lower) of fracture callus from young, adult and aged mice at 3 weeks after surgery. (D) Quantification of callus index (upper) and ratio of toluidine blue staining-positive versus callus area (lower) of fractured callus from young, adult and aged mice at week 3 after surgery. Results are presented as mean ± SD. Statistical analysis: One-way ANOVA with Tukey's multiple comparisons test. *P* values were directly labeled above the dot plots for identification of significance. (E) Representative immunohistochemistry results showing Cxcr4-positive cells in fracture callus of young, adult and aged mice at week 3 after surgery (n = 3 per group). (F) Representative immunofluorescent results showing Cxcr4 and CD51 co-expressing cells in fracture callus of young, adult and aged mice at week 3 after surgery (n = 3 per group).

All statistical calculations and graphs were created using GraphPad Prism 9. The results shown were reported as mean ± standard deviation (SD). When applicable, Student's t-test (Fig. 1, Fig. 2, Fig. 3B, and Supplementary Figs. 2B, 2D, 3F, 3H), one-way ANOVA (Fig. 1, Fig. 4D, and Supplementary Fig. 3B–D) or two-way ANOVA (Supplementary Fig. 4B) were used to evaluate the statistical significance. Logistic regression analysis were used to assess the linearity correlation between maximum load and SUVmax of radiotracers in Fig. 3C. Statistical significance was accepted as a p-value less than 0.05.

## 3 Results

### 3.1 Identification of CXCR4 as a marker and target of SSCs enabling CXCR4-targeted PET-CT monitoring

We first analyzed the interactions between CXCR4 protein and ligand Pentixafor to show the potential interactions between the protein and the radiotracer (Supplementary Fig. 1A). All functional residues were identified and classified according to their interactions. There are multiple groups of residues used to form interactions between receptor protein and ligand, such as the hydrogen bond formed by ASP182 of CXCR4 and ligand Pentixafor. With these interaction forces, the binding energy of protein-ligand complex was −11.1 kcal/mol, which is a satisfying performance. Indicate that Pentixafor binds to CXCR4, as previously reported [23,24,[45], [46], [47], [48]].

To identify this approach to target skeletal stem and non-stem stromal populations using isotope-labeled radioprobes, we isolated murine SSCs (Lin^-^6C3^-^Thy1.2^−^CD105^−^CD51^+^CD200^+^ subset, defined by Chan et al., 2018 [10]) and non-SSC subsets (NSCs, defined as Lin^-^6C3^-^Thy1.2^−^CD105^+^, Lin^-^6C3^-^Thy1.2^−^CD51^−^ and Lin^-^6C3^-^Thy1.2^−^CD105^−^CD51^+^CD200^−^subsets), from the fracture callus (fracture group, Fracture) and contralateral limbs (control group, Ctrl, Supplementary Fig. 1B–D) of mice undergoing an open femoral mid-diaphyseal fracture. Bulk RNA sequencing revealed distinct transcriptional signatures for SSCs, including high expression of canonical markers (e.g., *Cd51*) and proliferation-associated genes (*Mki67, Prc1, Top2a*), but not markers of differentiating or mature osteoblasts such as *Col1a1, Alpl, Atf4* and *Bglap2* (Fig. 1A).

Given the growing number of clinically approved PET tracers targeting cell surface receptors, we screened highly enriched SSC markers for compatibility with existing FDA-approved or radiotracers in III/IV phase clinical trials. *Cxcr4* emerged as a strong candidate, displaying significantly higher expression in SSCs than in NSCs (Fig. 1A). This pattern was validated using an independent neonatal SSC RNA-seq dataset (Fig. 1B) [19]. Notably, elevated *Cxcr4* expression was unique to long-bone SSCs, whereas vertebral SSCs showed minimal expression (Fig. 1C), indicating site-specific chemokine receptor specialization.

FACS-based gene expression analysis and immunofluorescence staining of the fracture callus confirmed co-localization of CXCR4 and the SSC marker CD51 (Fig. 1D and E). Further supporting the translational potential of this approach, retrospective review of a pediatric ^18^F-Pentixafor PET-CT scan revealed robust tracer uptake in the growth plate and metaphysis area, the region known to be rich in SSCs (Fig. 1F) [[49], [50], [51]]. We also performed cell binding assay to show the correlation between ^18^F-Pentixafor uptake and SSC amounts which suggests the binding between ^18^F-Pentixafor and SSCs, rather than NSCs whose amount increase does not lead to elevated ^18^F-Pentixafor uptake (Supplementary Fig. 1F). Of note, ^18^F-Pentixafor PET imaging can tell the difference between SSC and NSC at a relatively low cell amount (less than 10^4^ cells). Suggesting this technique is a precise imaging approach in evaluating the expression of CXCR4 on SSCs with a relatively small cell number.

Collectively, these findings demonstrate that SSCs express disproportionately high levels of *Cxcr4*, establishing a biological basis for CXCR4-targeted PET-CT imaging of SSCs.

### 3.2 Reduced ^18^F-Pentixafor uptake reflects age-related loss of SSCs in long bone metaphyses

Age-related SSC dysfunction is a major contributor to decreased bone formation and osteopenia [16]. To determine whether ^18^F-Pentixafor PET-CT can capture SSC changes associated with aging, we compared tracer uptake in the metaphysis of young (1-month-old) and aged (24-month-old) mice. Young mice exhibited strong ^18^F-Pentixafor signal in metaphyseal regions, whereas aged mice displayed substantially reduced uptake, consistent with closure of the growth plate and diminished SSC pools (SUV_max_ Young vs. Aged, 0.6723 ± 0.0509 vs. 0.2953 ± 0.0352, Fig. 2A–C) [15,51,52]. Flow cytometry confirmed a significant reduction in SSC abundance in aged long bones (Fig. 2D and E). Additionally, SSCs from aged mice exhibited diminished differentiation and mineralization capacity, accompanied by reduced *Cxcr4* expression (Fig. S2A–B). Micro-CT analysis further revealed osteopenic changes in aged femurs (Fig. S2C–E).

To assess translational relevance, we retrospectively analyzed ^18^F-Pentixafor PET-CT scans from adults evaluated for adrenal lesions but without skeletal abnormalities (Table 2). Since whole-body scans were rarely available, we quantified uptake in the femoral head, a region representative of a long bone metaphysis. Similar to murine findings, ^18^F-Pentixafor uptake was significantly lower in aged individuals compared with younger adults (Fig. 2F and G). These findings indicate that ^18^F-Pentixafor PET-CT sensitively reflects age-related reductions in SSC abundance and function, highlighting its potential utility in evaluating age-associated bone loss.

**Table 2:** Baseline characteristics of^18^F-Pentixafor PET-CT cohorts.

Variable	Adult	Aged
Male	N = 6	N = 11
Female	N = 8	N = 12
Age, median (interquartile range), years	30(21-40)	65(61-83)
SUV_max_ of femoral head area, median (interquartile range)	0.49(0.13-0.98)	0.29(0.08-0.48)
		

### 3.3 CXCR4 levels correlates with SSC function and a CXCR4-targeted radiotracer correlates with biomechanical outcomes in murine fracture model

To assess whether CXCR4 signaling function influences SSC osteogenic potential and migration activity, we exposed isolated murine SSCs and treated SSCs with CXCR4 agonist (NUCC-390) or antagonist (Plerixafor) (Supplementary Fig. 3A). Low-dose agonist treatment (0.63-1.25 μM NUCC-390) significantly enhanced SSC differentiation, mineralization and migration (Supplementary Fig. 3B and 3D), while CXCR4 inhibition (0.63-2.5 μM Plerixafor) impaired these processes (Supplementary Fig. 3C and 3D). To evaluate the *in vivo* relevance of these findings, we locally administered Plerixafor (2 mg/kg) into the fracture site of murine models. ^18^F-Pentixafor PET-CT imaging at 3 weeks post-surgery showed significantly reduced uptake in antagonist-treated fractures, accompanied by delayed callus formation. Micro-CT analysis confirmed reduced bone volume in the fracture callus (Supplementary Fig. 3E–F), and histological assessments revealed diminished cartilage content and fewer Cxcr4^+^ cells (Supplementary Fig. 3G–H). Together, these results demonstrate that CXCR4 activity is essential for SSC-mediated fracture repair.

We next tested whether ^18^F-Pentixafor uptake could predict fracture healing outcomes [25,26,33,53]. Early inflammatory responses during the first postoperative week can confound CXCR4-targeted imaging due to immune cell infiltration (left of Fig. S4A) [54,55]. However, at 2-3 weeks post-fracture, ^18^F-Pentixafor uptake localized predominantly to the callus, with minimal background uptake in soft tissues (middle, right of Supplementary Fig. 4A and Supplementary Fig. 4B). Furthermore, through biodistribution studies, we confirmed that the fracture callus displayed relatively high ^18^F-Pentixafor uptake (Supplementary Fig. 4C). Thus, after the initial acute inflammatory stage, ^18^F-Pentixafor signal persists in fracture callus, in line with murine fracture healing taking place over 3-5 weeks [36].

Since current radiotracers utilized for PET-CT evaluation of bone diseases largely rely on ^18^F-FDG and ^18^F-NaF, we sought to compare the performance of ^18^F-Pentixafor to these established benchmarks in the setting of both fracture healing and critical size defect models 3 weeks after surgery. Comparing fracture healing with critical-sized defects that by definition are unable to heal (Supplementary Fig. 4D), we found that ^18^F-Pentixafor uptake in the callus area only in the healing fracture, with robust differentiation between healing and non-healing fractures (Fig. 3A and B). In contrast, ^18^F-FDG and ^18^F-NaF did not exhibit significant differences in radiotracer uptake between healing and non-healing fractures.

To assess the biomechanical property of fractured bone, we further utilized a 3-point-bending test at 4 weeks after surgery (Supplementary Fig. 4E) to evaluate the maximum load the callus can sustain, which reflects the primary outcome of fracture healing. Intriguingly, the SUV_max_ of ^18^F-Pentixafor showing strongly correlated with mechanical strength of fractured bone measured by three-point bending (R^2^ = 0.7695, Fig. 3C, left), outperforming ^18^F-NaF (R^2^ = 0.1431, Fig. 3C, right) and ^18^F-FDG (R^2^ = 0.0001, Fig. 3C, middle). ^18^F-Pentixafor uptake was also positively correlated with mineralized callus volume (Fig. 3D).

Taken together, these results demonstrate that CXCR4-targeted ^18^F-Pentixafor PET-CT provides biologically relevant prognostic information, reflecting SSC-driven osteogenesis and predicting fracture mechanical competence.

### 3.4 Decreased ^18^F-Pentixafor uptake in the aged fracture callus corresponds to reduced Cxcr4^+^ SSCs and impaired fracture healing

To further determine whether ^18^F-Pentixafor uptake captures age-related impairments in fracture healing, we evaluated fractures in young, adult, and aged mice. ^18^F-Pentixafor uptake at 3 weeks post-surgery was highest in young mice and lowest in aged mice, mirroring the severity of healing deficits (Fig. 4A and B). Histological analyses revealed reduced callus index and diminished cartilaginous callus formation in aged animals (Fig. 4C and D).

Importantly, reduced tracer uptake corresponded closely with decreased *Cxcr4* expression in the fracture callus and reduced CXCR4/Cd51 co-localization (Fig. 4E and F). Thus, decreased ^18^F-Pentixafor uptake is seen in aged mice in concert with reductions in fracture induced SSCs expansion, supporting the utility of ^18^F-Pentixafor for assessing delayed or compromised healing.

### 3.5 Clinical case: ^18^F-pentixafor-based PET scans assist in evaluating hamate fracture healing and clinical decision in a young patient

We further explored the clinical feasibility of CXCR4-targeted imaging in an 18-year-old female patient with hamate fracture, a rare carpal bone fracture but with uncertain outcomes after conservative treatment, including casting followed by splinting [[56], [57], [58]]. Six months post-injury after an accident, the patient continued to experience pain and functional impairment. Although her orthopedic surgeon recommended surgical treatment, the patient decided on conservative treatment. ^18^F-Pentixafor PET-CT was applied to evaluate the osteogenic capacity as well as SSC amounts in light of the patient preference for conservative management.

**Fig. 5:**
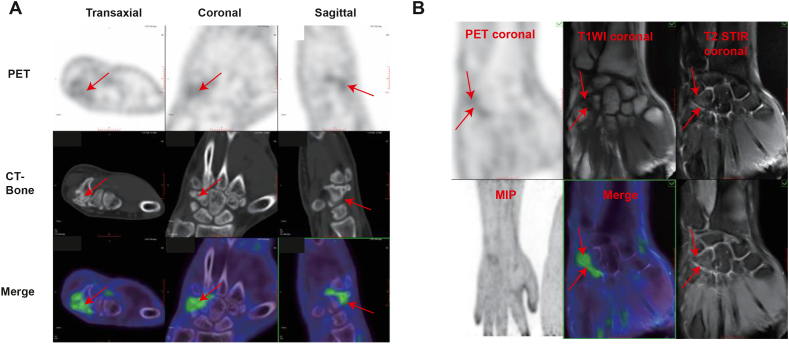
Clinical Case: ^18^F-Pentixafor-based PET scans assist in evaluating hamulus fracture healing in a young patient (A) ^18^F-Pentixafor PET-CT results of an 18-year-old female patient suffering hamulus fracture 6 months after a right palm injury (after 6 months), showing ^18^F-Pentixafor uptake in fracture line and callus area. Scale bar = 10 mm. (B) ^18^F-Pentixafor PET-MR results of the same patient, showing ^18^F-Pentixafor uptake in fractured bone with healed cortical bone and bone marrow edema (T1WI: T1-weighted imaging, STIR: Short Time of Inversion Recovery, MIP: Maximum Intensity Projection). Scale bar = 10 mm.

^18^F-Pentixafor PET-CT revealed pronounced tracer uptake localized to the fracture line and callus (SUV_max_ 4.6, Fig. 5A), indicating active bone regeneration and SSC accumulation. Based on these findings and after consulting her orthopaedic surgeon, conservative management was continued. At 1.5 years post-injury, a repeat ^18^F-Pentixafor PET-MR demonstrated healing cortical bone with residual-modest marrow edema and reduced but persistent tracer uptake at the prior fracture line, consistent with ongoing remodeling (SUV_max_ 3.2, Fig. 5B). This case suggests the correlation of ^18^F-Pentixafor uptake and fracture healing outcomes, which parallel at least in part our findings in preclinical models. In this case, ^18^F-Pentixafor-based PET/CT provided prognostic information with the potential to assist clinical decision-making in a complex fracture healing scenario.

## 4 Discussion

In this study, we identify CXCR4-targeted ^18^F-Pentixafor PET–CT as a sensitive, noninvasive imaging strategy capable of visualizing SSC abundance and functional activity during bone formation, aging, and fracture repair. By integrating transcriptomics, mechanistic perturbation, multi-modal imaging, and translational human data, we demonstrate that CXCR4 expression is selectively enriched in long-bone SSCs, dynamically reflects their osteogenic capacity, and can serve as a predictive biomarker for fracture-healing outcomes. Together, these findings introduce a previously unrecognized application of CXCR4-directed molecular imaging in skeletal biology and orthopaedic medicine.

CXCR4 is markedly enriched in SSCs compared with non-stem skeletal stromal populations, as observed by both bulk RNA sequencing of FACS-isolated populations, and immunostaining of the fracture callus. Importantly, CXCR4 expression was substantially higher in long-bone SSCs compared with vertebral SSC lineages [19], highlighting a previously unappreciated site-specific pattern of chemokine-receptor expression within skeletal progenitor niches. Functionally, CXCR4 is not merely an SSC marker, as CXCR4 agonist stimulation enhanced SSC osteogenic differentiation and mineralization, whereas CXCR4 antagonists impaired osteogenesis and delayed fracture repair. Mechanistically, these data also support a model in which CXCR4 not only guides progenitor localization but also regulates lineage progression during bone formation. These findings extend prior work implicating CXCR4 in skeletal cell migration and niche retention, here examining the role of CXCR4 for the first time in SSCs [22,59,60].

Previous studies have highlighted the role of CXCR4 in hematopoietic stem cell homing and immune cell trafficking [21,[61], [62], [63]], and its utility as a target for molecular imaging using ^68^Ga–or ^18^F-labeled ligands such as Pentixafor in various malignancies and inflammatory conditions [23,24,45,64,65]. However, relatively few studies have examined CXCR4-targeted imaging within the context of skeletal biology. Our results show that ^18^F-Pentixafor uptake reflected CXCR4 expression *in vivo*. Uptake was robust in SSC-rich metaphyseal regions of young mice but diminished markedly with aging, paralleling the well-documented age-related decline in SSC abundance and function. Translational analyses of human PET-CT scans revealed a similar age-related decrease in femoral-head uptake, demonstrating that CXCR4-targeted molecular imaging can capture clinically relevant variation in skeletal regenerative potential. These results align with seminal studies showing that aging diminishes SSC number, impairs osteochondral differentiation [16], and contributes to osteopenia, our data extend these findings by providing a non-invasive imaging surrogate for these cellular deficits.

Among the most compelling findings is that ^18^F-Pentixafor uptake at 3 weeks post-fracture predicted callus maturation, mineralized callus volume, and mechanical strength, suggesting that the signal from this probe provides a holistic sense of the outcome of fracture healing. Traditional imaging approaches failed to predict the outcome of fracture healing due to their exclusive focus on bone architectural parameters at the time of imaging. Thus, having access to an imaging approach that reflects the functional capacity of skeletal progenitors mediating bone formation would fill an important gap and may ultimately provide critical information to guide clinical management. We propose that such an approach would be useful to apply in settings where there is a clinical concern for potential non-union to aid in assessing the non-union risk and accordingly tailoring clinical management. However, further clinical validation will be needed to translate this model into clinical practice.

Compared with traditional bone radiotracers, ^18^F-Pentixafor distinguished non-critical fractures from critical-size defects, correlated strongly with biomechanical outcomes, and outperformed both ^18^F-FDG (metabolic imaging) and ^18^F-NaF (mineralization imaging). This suggests that imaging SSC activity provides earlier and more biologically relevant prognostic information than measuring glucose metabolism or mineral deposition alone [31,66]. Moreover, aged mice displayed both reduced ^18^F-Pentixafor uptake and impaired callus formation, linking poor repair capacity directly to aging associated reduction in the functional capacity and/or numbers of CXCR4^+^ SSCs. Our clinical case implies that the ^18^F-Pentixafor PET-CT approach developed here will have real-world translational utility in guiding clinical decision in the context of bone fracture management. However, future clinical studies relating ^18^F-Pentixafor PET-CT signal to fracture healing outcomes will be needed to validate this approach. If this is validated, then ^18^F-Pentixafor PET-CT may aid in providing early and more accurate identification of patients at risk of non-union, thereby identifying patients that may warrant targeted surgical or medical interventions.

## 5 Limitations

Notably, our results do not supersede the value of traditional approaches to evaluate mineralization and bone metabolism in skeletal disorders. Several limitations of this study should be acknowledged. First, although CXCR4 is enriched in SSCs, it is not exclusively expressed by them, as inflammatory and hematopoietic cells also express CXCR4. We here minimized this confounding by imaging after the acute inflammatory phase. However, there are likely opportunities to further refine this approach, such as through the use of dual-tracer imaging or SSC-specific conditional models, which would allow for greater insight into the specific cells mediating the ^18^F-Pentixafor signal seen here. Second, PET resolution does not permit direct visualization of individual progenitor niches, thus, uptake reflects collective tracer accumulation within a region rather than discrete SSC pools. Third, our human data were retrospective and limited to anatomical regions incidental to clinical scans, prospective trials will be essential to confirm diagnostic performance across fracture types and patient populations. Last but not least, CXCR4 is not itself a purely SSC-specific marker, raising the possibility that in some scenarios non-SSC derived signal may confound interpretation of ^18^F-Pentixafor PET-CT for bone healing applications.

## 6 Conclusion

In summary, our work establishes CXCR4-targeted ^18^F-Pentixafor PET-CT as a robust, noninvasive modality for imaging SSC abundance and function, providing the first imaging method to directly assess SSC numbers or function. Since ^18^F-Pentixafor has been widely utilized for other indications, repurposing it for skeletal applications may accelerate clinical translation. By enabling early and accurate assessment of regenerative potential, this imaging strategy provides a non-invasive approach that holds promise for enabling more precise diagnosis, risk stratification, and personalized treatment of traumatic skeletal disorders.

## Author contributions

Conceptualization: X.S., M.B.G and Z.L.

Methodology, validation, investigation, resources and data analysis: Z.L., D.Z., D.B., J.H., Z.X., Q.X., L.T., GL.W., GF.W., P.W.

Writing-Original manuscript: X.S., M.B.G and Z.L.

Supervision and Funding acquisition: X.S., M.B.G. and Z.L.

## Ethics

All experiments involving animals were conducted according to the ethical policies and procedures approved by the Institutional Animal Care and Use Committee (IACUC) of the First Affiliated Hospital, Zhejiang University School of Medicine (Approval No. 2023-986) and following ARRIVE (Animal Research: Reporting of *In vivo* Experiments) guidelines.

All experiments involving patients were conducted according to the ethical policies and procedures in accordance with the ethical standards of the Helsinki Declaration of 1975 and were approved by the Clinical Research Ethics Committee of the First Affiliated Hospital, Zhejiang University School of Medicine (Approval No. IIT20250082C-R1). Approved clinical trials were registered on ClinicalTrials.gov. and ID: NCT07122674.

## Declaration of generative AI in scientific writing

No generative artificial intelligence (AI) or AI-assisted technologies were used in the preparation of this manuscript.

## Funding statement

This work was supported by grants from the 10.13039/501100012166National Key Research and Development Program of China (2023YFF0716000, 2024YFC2419800), 10.13039/501100001809National Natural Science Foundation of China (82472013, 82071965, 82502852), Zhejiang Province Vanguard Plan Project (2025C01111), Major plan of Jointly Constructed project by the Science and Technology Department of the 10.13039/501100005891State Administration of Traditional Chinese Medicine and the 10.13039/501100012175Zhejiang Provincial Administration of Traditional Chinese Medicine (GZY-ZJ-KJ-24025) and National High Performance 10.13039/501100015702Medical Device Innovation Center Innovation Fund (NMED2024CX-01-002) for X.S. and 10.13039/501100001809NSFC Youth Fund (82502852) for Z.L. 10.13039/100010493MBG is supported by 10.13039/100014276Pershing Square Sohn Cancer Research Alliance and Pershing Square MIND Prize awards.

## Conflicts of interest statement

The authors have no conflicts of interest relevant to this article.

## Data Availability

Z.L., J.H., and all corresponding authors can provide all information about the data presented in the article and all data are available upon reasonable requests.
