# Supplementary material for: A novel non-invasive approach monitoring skeletal stem cell function through 18F-Pentixafor PET-CT
Source: J Orthop Translat. 2026 May 20;58:101129. doi: 10.1016/j.jot.2026.101129 (PMC13214306; doi:10.1016/j.jot.2026.101129)
Supplement: Multimedia component 1 [file mmc1.docx]

**Supplementary materials**


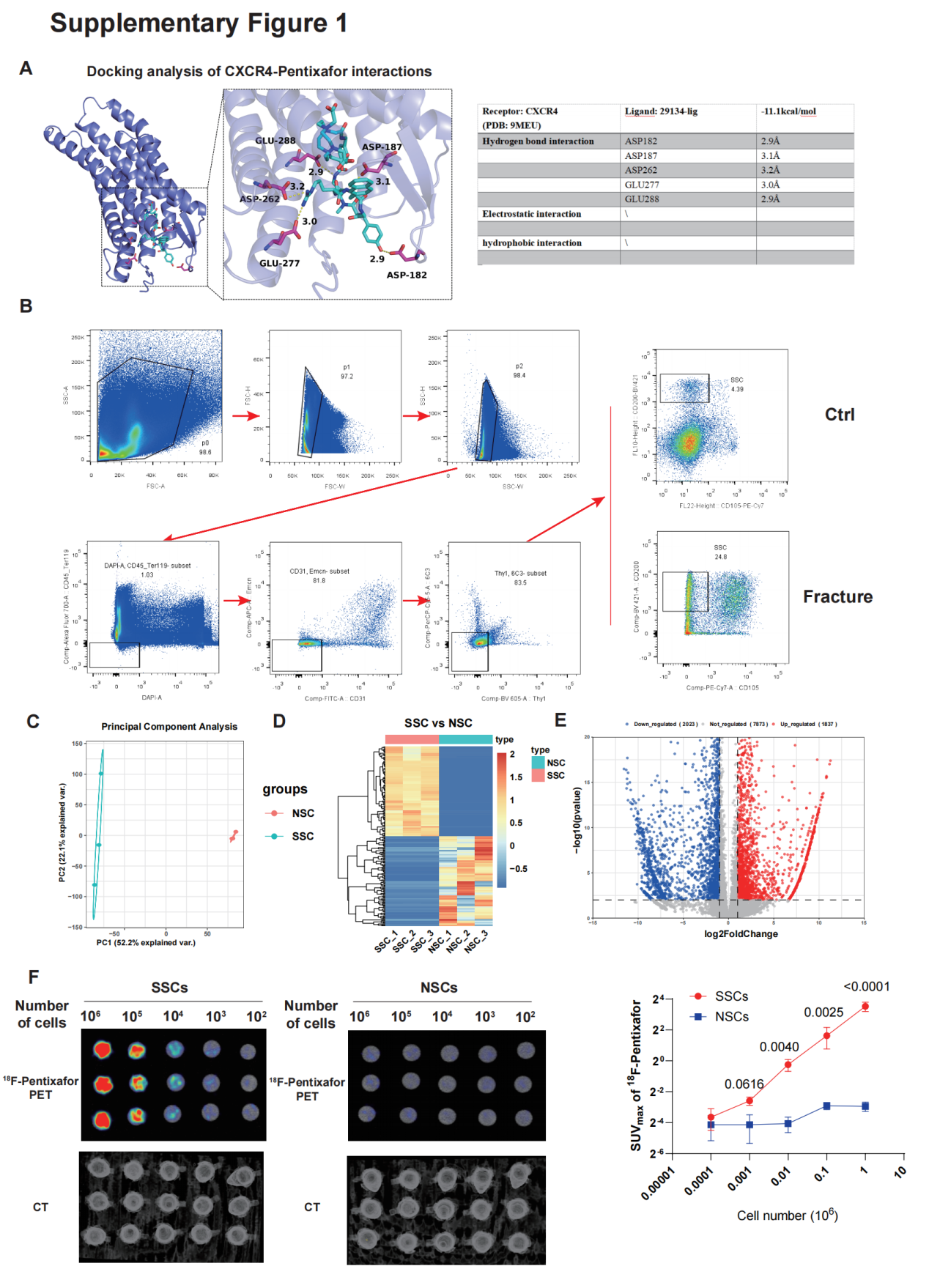


**Supplementary Figure 1**

1. Docking analysis showing the interactions between CXCR4 and Pentixafor as well as the protein-ligand binding energy.
2. Representative flow results showing gating strategy of SSCs from fracture callus (Fracture) and contralateral limb (control, Ctrl).
3. Principle component analysis of SSCs and NSCs cell groups (n=3).
4. General differential gene heatmap of SSCs and NSCs (n=3).
5. Volcano plot showing up-regulated and down-regulated genes in SSCs compared to NSCs.
6. Cell binding assay showing the correlation between ^18^F-Pentixafor uptake and the amount of SSCs. Cell amounts were labeled in X axis and P value (Two-way ANOVA) were directly labeled above each timepoint. (n=3)


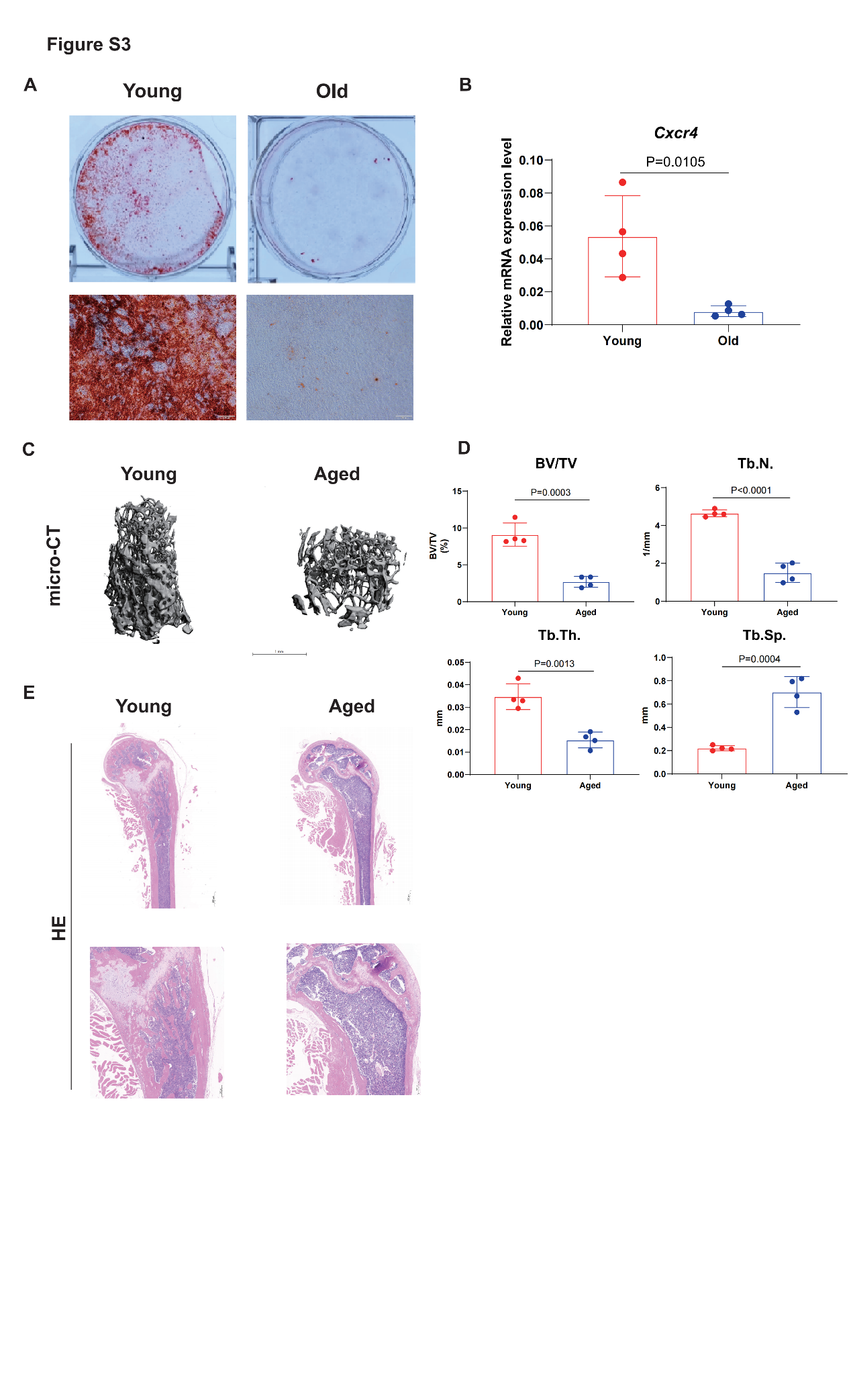
**Supplementary Figure 2**

1. Representative images of Alizarin Red Staining results of isolated, cultured and differentiated SSCs from young and aged mice, and (B) qPCR results of Cxcr4 in SSCs from young and aged mice (n=4 per group).
2. Representative μCT images of femoral metaphysis of young and aged mice and (D) analysis of bone volume/total volume (BV/TV), trabecular number (Tb.N.), trabecular thickness (Tb.Th.) and trabecular spacing (Tb.Sp.) and cortical thickness (Ct.Th.) from young and aged mice (n=4 per group).

(E) Representative H&E images of femoral metaphysis of young and aged mice. Results (Supplementary Fig. 2B and 2D) are presented as mean ± SD. Statistical analysis: Student’s t-test. *P*-values are directly labelled on the plot showing significance.


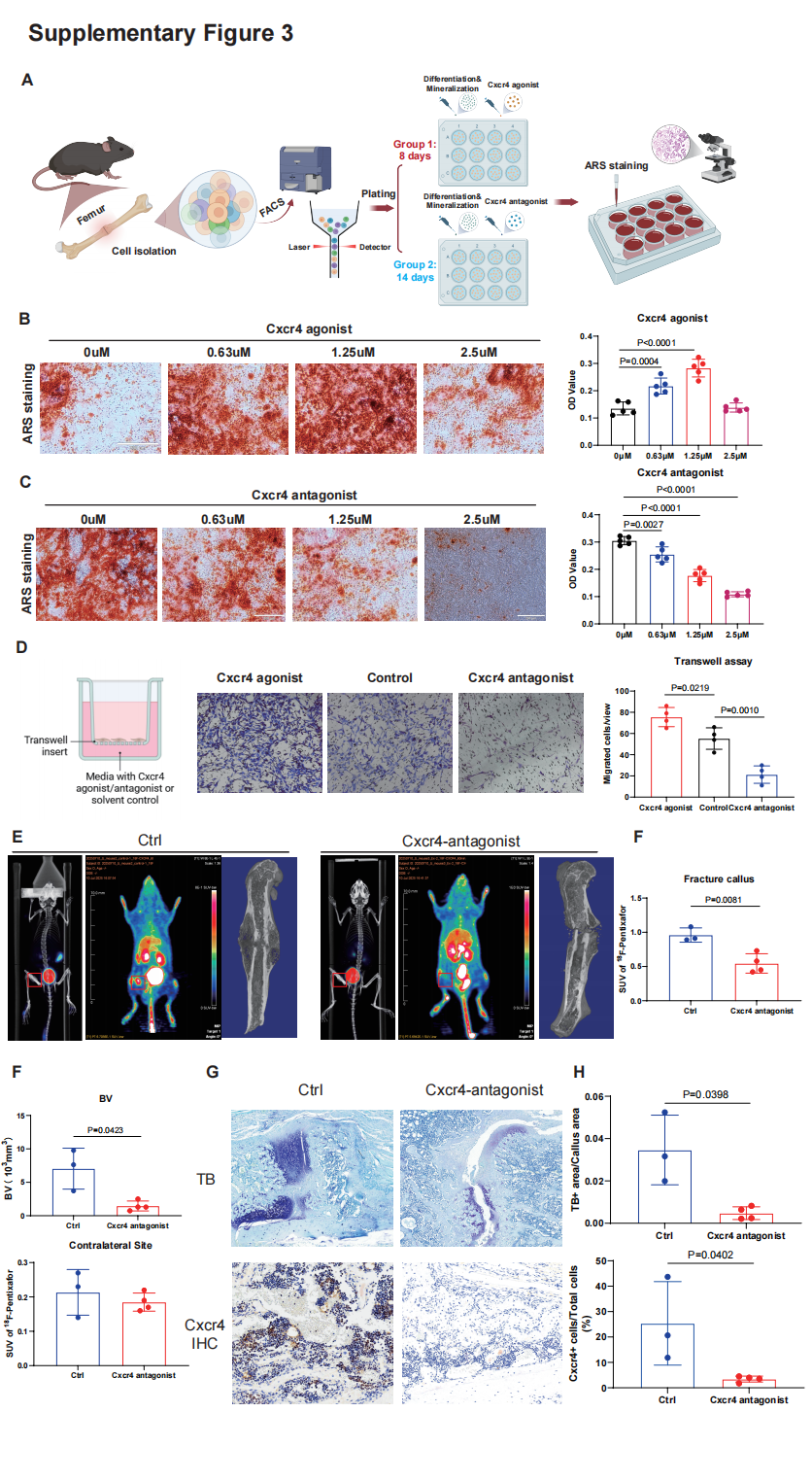


**Supplementary Figure 3**

(A) Graphical illustration of the methods used to assess the role of Cxcr4 in the differentiation and mineralization capacity of SSCs.

(B) Representative ARS staining and Alizarin Red Assay results showing the differentiation and mineralization capacity of SSCs stimulated with Cxcr4 agonist, NUCC-390 (n=5 per group, Results are presented as mean ± SD. Statistical analysis: One-way ANOVA with Tukey’s multiple comparisons test).

(C) Representative ARS staining and Alizarin Red Assay results showing differentiation and mineralization capacity of SSCs stimulated with Cxcr4 antagonist, Plerixafor (n=5 per group, Results are presented as mean ± SD. Statistical analysis: One-way ANOVA with Tukey’s multiple comparisons test).

(D) Graphical illustration and transwell assay showing SSCs stimulated with a Cxcr4 agonist, a Cxcr4 antagonist or a vehicle control (n=4 per group).

1. Representative PET-CT and micro-CT results showing ^18^F-Pentixafor uptake in fracture callus and longitudinal axis of the fractured femur from Cxcr4-antagonist treated (Cxcr4-antagonist) and control mice (Ctrl). (n=4 for Cxcr4-antagonist treated mice and n=3 for control group. Scale bar shown as labeled.)

(F) Quantification of micro-CT results from fractured bone from Cxcr4-antagonist treated (Cxcr4-antagonist) and control mice (Ctrl). (n=4 for Cxcr4-antagonist treated mice and n=3 for control group). Results are presented as mean ± SD. Statistical analysis: Student’s t-test. *P*-values are directly labelled on the plot showing significance.

(G) Representative toluidine blue staining showing microstructure of fracture callus area.

(H) Quantification of Toluidine blue-positive area/Total area and Cxcr4-positive cells in fracture callus area. Results are presented as mean ± SD. Statistical analysis: Student’s t-test. *P*-values are directly labelled on the plot showing significance.


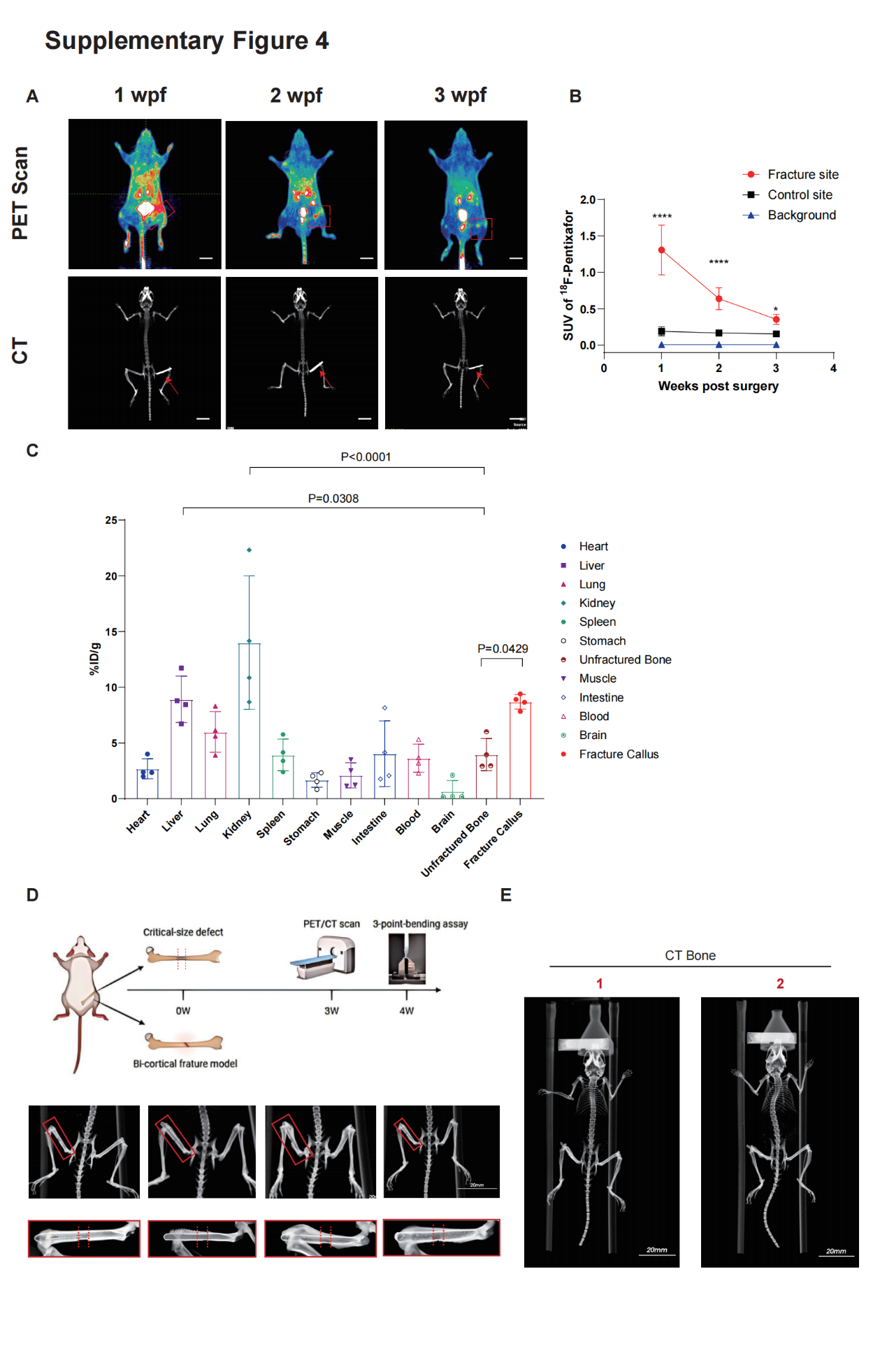


**Supplementary Figure 4**

(A) Representative ^18^F-Pentixafor PET-CT of murine fracture model at different timepoints after surgery (wpf: week-post-surgery). (B) SUV_max_ of ^18^F-Pentixafor at the fracture site (fracture callus), control site (contralateral femoral diaphysis) and background uptake. (C) Biodistribution of ^18^F-Pentixafor uptake in crucial organs including heart, liver, lung, kidney, spleen, stomach, unfractured bone, muscle, intestine, blood cells, brain and fracture callus. (D) Graphical illustration of the bi-cortical fracture model and critical-sized defect model and timepoint of following PET/CT scan as well as three-point bending assays. The dashed lines in the lower row represent the critical-sized defect depot. (E) CT scan of bone showing two murine fracture models representing distinct biomechanical outcomes related to Fig. 4C-D.
